# Valorization of the essential oil from *Drypetes gossweileri* S. Moore (Putranjivaceae): *in vitro, in vivo*, and *in silico* nematicidal activity

**DOI:** 10.3389/fpls.2023.1260360

**Published:** 2023-11-29

**Authors:** Jean Pierre Mbula, Maria Fe Andres, Emmanuel M. Kitete, N. G. Kasiama, D. D. Tshilanda, K. N. Ngbolua, D. S. T. Tshibangu, O. Onautshu, Azucena González-Coloma, Pius T. Mpiana

**Affiliations:** ^1^ Faculté des Sciences, Université de Kisangani, Kisangani, Democratic Republic of Congo; ^2^ Instituto de Ciencias Agrarias, Consejo Superior de Investigaciones Científicas, Madrid, Spain; ^3^ Faculté des Sciences, Université de Kinshasa, Kinshasa, Democratic Republic of Congo

**Keywords:** *Drypetes gossweileri*, essential oil, insect antifeedant, nematicidal activity, phytotoxic, molecular docking, Kisangani

## Abstract

The chemical composition, insect antifeedant, *in vtro/in vivo* nematicidal activity, phytotoxicity, and *in silico* nematicidal activity of the essential oil (EO) of the African medicinal plant *Drypetes gossweileri* were studied. Chemical analysis using GC/MS indicated that benzyl isothiocyanate (96.23%) was the major compound, followed by benzyl cyanide (1.38%). The biocidal effects of this oil were tested against insect pests and root-knot nematodes. All the insect species tested were significantly affected by the oil according to their feeding adaptations (*Spodoptera littoralis* and *Myzus persicae* were less affected than *Rhopalosiphum padi*) with efficient doses (EC_50_) of 29.4 8.3 μg/cm^2^, 14.744 8.3 μg/cm^2^, and 8.3 μg/cm^2^, respectively. The oil was highly effective against juveniles J2 of the nematode *Meloidogyne javanica*, with LC_50_–LC_90_ values of 0.007 mg/mL–0.0113 mg/mL. *D. gossweileri* EO at minimum lethal concentrations (MLC) and below strongly inhibited egg hatching *in vitro*, whereas soil treatment caused a strong suppression of nematode population, infection frequency, and multiplication rate. The EO inhibited ryegrass (*Lolium perenne*) germination at 0.4 mg/mL, while at 0.1 mg/mL, its effects on germination, root and leaf growth were moderate (32.4%, 8.4%, and 18.3%, respectively). The tomato (*Solanum lycopersicum*) germination was not affected by the EO, but the root growth was reduced (56% at 0.1 mg/mL) at a dose 10 times higher than the LD_50_ calculated for *M. javanica* J2 mortality. Molecular docking of the nematicidal effects of the oil using PyRx revealed a strong interaction between potassium chloride transporting KCC3 (PDB ID: 7D90) and benzyl cyanide at a distance of 2.20 A° with GLN C:350, followed by benzyl isothiocyanate at a distance of 2.78 A° with ARG B:294. The *in vivo* nematicidal effects of *D. gossweileri* EO on *M. javanica* penetration and reproduction in tomato roots further support the potential of this EO as a nematicidal agent with insect antifeedant effects, which could be used by local farmers for crop protection.

## 1 Introduction

Worldwide, agriculture has problems related to soil fertility, especially to pests and diseases that significantly contribute to annual losses in crop yield (FAO, 2020). The application of chemical pesticides is an effective control method for plant pests and diseases that increase agricultural production but represents a serious threat to public health and the environment (Bonner and Alavanja, 2017). This problem has affected African agricultural systems worldwide.

The African population is estimated at 1.3 billion and is expected to double by 2050 (Searchinger et al., 2019), compromising African food production systems with low productivity. Agricultural pesticides can increase crop production, improve nutrition, raise household income, facilitate local and international trade, and control mosquitoes in Africa. However, the use of pesticides in Africa has polluted freshwater sources (Nesser et al., 2016; Osoro et al., 2016), endangered wildlife (Ogada, 2014), and beneficial organisms (Vak and Koomen, 2013), and pesticide residues greater than the recommended daily intake have been found in cooked food samples (Ogah, 2012). In addition, European green agriculture standards have created trade barriers for African farmers. In this context, new safe biopesticides are needed to improve crop protection, food safety, and trade, while being locally available and sustainable (Fortuna, 2022).

Plant-based biopesticides, including compounds, extracts, and essential oils, are an important group of safer products for pest management. Their chemical composition is complex limiting the development of resistance by target pests. Therefore, plant-based biopesticides are promising and safer crop protection alternatives with unique chemical compositions and modes of action (Khursheed et al., 2022).

Species of the genus *Drypetes* of the Putranjivaceae family are used in traditional medicines in subSaharan Africa and Asia, and some are used against pests (Wansi et al., 2016). *Drypetes gossweileri* S. Moore is a medium-sized dioecious (30 m–42 m in height; bole straight, up to 120 cm in diameter) species found in the Democratic Republic of Congo (DRC), Gabon, Equatorial Guinea, Central African Republic, and Cameroon. The bark is used as an analgesic for venereal diseases, urethral problems, reptile-repellent, fish poisoning, anthelminthic, vermifugal, and antifilarial (Wansi et al., 2016), and to protect stored food against pests (Aba et al., 2013). The essential oil of *D. gossweileri* is known for its antimycobacterial (Moni et al., 2018), antimicrobial (Moni et al., 2018; Graf and Stappen, 2022), and antifungal activities (Ghislaine et al., 2013).

Phyochemical studies of *D. gossweileri* extracts have shown the presence of friedelanes, oleananes, diterpenoids, and phenylethanoids (Wansi et al., 2016). Glucosinolates (GSLs) such as 4-hydroxybenzyl-glucosinolate and benzyl glucosinolate have been identified in the bark of *D. gossweileri* (Montaut et al., 2017). The essential oil (EO) from this plant species contains isothiocyanates (ITCs), benzyl isothiocyanate (Ndoye Foe et al., 2016), and benzyl cyanide (Agnaniet et al., 2003) as major components, which originate from hydrolysis of their precursor GSLs. Glucosinolates (GSL, β-thioglucoside-N-hydroxysulfates) are the precursors of isothiocyanates and are converted to ITCs after plant tissue damage by the action of the enzyme myrosinase. Glucosinolates and isothiocyanates are widely recognized as defensive compounds against generalist herbivores, are involved in host plant recognition by specialist predators (Hopkins et al., 2009), and have nematicidal properties (Wu et al., 2011; Ntalli and Caboni, 2017). Most of these compounds have been associated with many beneficial effects on human health, such as anticancer, antibacterial, antidiabetic, anti-obesity, antifungal, antioxidant, and antimutagenic activities (Di Gioia et al., 2020).

As part of an ongoing project exploring the pesticidal potential of African medicinal plants for local farmers, we assessed the essential oil obtained from *D. gossweileri* sourced from the Kisangani region in the Democratic Republic of the Congo (DRC). This study aimed to determine its efficacy as a biopesticide against various insect pests (*Rhopalosiphum padi*, *Myzus persicae*, and *Spodoptera littoralis*) and a phytoparasitic nematode (*Meloydogine javanica*) of economic importance. The moth *S. littoralis* (Egyptian cotton leafworm) feeds on a wide range of horticultural crops (Muñoz-Rodríguez et al., 2018). The aphid *M. persicae* affects many plant species with a particular affinity for Brassicaceae crops (Kim and Jander, 2007). The cereal aphid *R. padi* is a major pest of cereals (Greenslade et al., 2016). The root-knot nematode, *M. javanica*, which is present in temperate and tropical regions, is the most destructive nematode because of the formation of galls in the host roots. In the DRC, *M. javanica* attacks several crops (beans, soybeans, and tomatoes), contributing to more than 5% of crop loss (Chaudhary et al., 2019; Alekcevetch et al., 2021).

In this work, the phytochemical composition of *D. gossweileri* essential oil from the Kisangani region (DRC) has been studied along with its bioactivity against *S. littoralis*, *M. persicae*, *R. padi*, and *M. javanica*. We also tested the phytotoxicity of this EO on ryegrass (*Lolium perenne*) and tomato (*Solanum lycopersicum*) plants. The most significant effect was nematicidal activity. Molecular docking was used to study the major EO involved in this nematicidal effect.

## 2 Material and methods

### 2.1 Plant material

*D. gossweileri* (Putranjivaceae) leaves were collected from the equatorial evergreen rainforest of Masako Forest Reserve, near Kisangani City, Tshopo Province, Democratic Republic of the Congo (DRC). The reserve is situated near the equatorial line and has an elevation of approximately 500 m above the sea level. Its geographic coordinates are 0°36’N, 25°13’E, covering an area of 2105 Ha (Mbula et al., 2015).

### 2.2 Essential oil and chemical characterization (GC–MS analysis)

The essential oil of *D. gossweileri* was obtained by hydrodistillation of fresh leaves (100 g), extracted by hydrodistillation for 3 h using a Clevenger apparatus, dried on ammonium sulfate, and stored at 4°C until use.

The essential oil was analyzed by gas chromatography (GC) using a Shimadzu 2010 instrument and gas chromatography–mass spectrometry (GC–MS) equipped with a mass spectrometer Shimadzu GCMS-QP2010-Ultra Mass Detector (electron ionization, 70 eV, Kyoto, Japan). The carrieigas was Helium (50.0 kPa, 21.2 - max 25 mL/min total flow, 0.87 mL/min column flow). The capillary column was a Teknokroma TRB (95%) dimethyl (5%) dimethylpolysiloxane (30 m × 0.25 mm ID and 0.25 µm phase thickness). The working conditions were as follows: split ratio (20:1); injector temperature, 300°C; column temperature, 70°C –290°C for 6 min; staying at 290°C for 15 min; temperature of the transfer line connected to the mass spectrometer, 250°C; and ionization source temperature, 250°C. The injection consisted of 1 µL of the sample at 4 mg/mL in dichloromethane (two injections/sample). Compounds were identified by comparing the mass spectra with those available in the Wiley 229 and NIST Mass Spectral Databases, while relative area% was used for quantification of all the peaks obtained in the chromatograms, as previously described (Ruiz-Vasquez et al., 2022).

### 2.3 Antifeedant activity

For the antifeedant activity tests, insect colonies were kept at the ICA-CSIC. *Myzus persicae* and *Rhopalosiphum padi* were raised on bell pepper (*Capsicum annuum*) and barley (*Hordeum vulgare*) plants, respectively, and *Spodoptera littoralis* on artificial diet. The colonies were maintained at 22 ± 1°C, >70% relative humidity, and 16:8 h (L:D) photoperiod in a growth chamber.

Antifeedant bioassays were conducted using leaf disks or fragments (1.0 cm^2^) from *C. annuum* (*M. persicae, S. littoralis*) or *H. vulgare* (*R. padi*). The test substances were applied at an initial dose of 5 mg/mL (50 µg/cm^2^) and 10 μL of the solution was applied to the upper surface of the leaf samples.

For the aphids, 20 ventilated plastic boxes (2 cm × 2 cm) were used, each containing 10 apterous aphid adults (24 h–48 h old), and the aphids were allowed to feed in a growth chamber for 24 h under the same environmental conditions as above. For *S. littoralis*, six Petri dishes with two sixth-instar larvae each (>24 h after molting) were allowed to feed until 75% larval consumption of the control or treatment disks. Each experiment was repeated twice (SE <10%). Aphid settling was determined by counting the number of aphids on each leaf fragment. Feeding was calculated by measuring the disk surface consumption (digitalized with https://imagej.nih.gov/ij/, accessed on 23 October 2023). Settling/feeding inhibition (%FI or %SI) was calculated as %FI/%SI = [1 − (T/C) × 100], where T and C represent the settling/consumption of treated and control leaf fragments, respectively. The antifeedant effects (%SI/%FI) were statistically analyzed using a nonparametric Wilcoxon signed-rank test. Extracts and compounds with SI >70% were further subjected to dose–response experiments (three to five serial dilutions) to calculate their relative potency (EC_50_, the effective dose causing a 50% settling/feeding reduction) using linear regression analysis (%FI/SI on Log-dose) (Ruiz-Vasquez et al., 2022).

### 2.4 Nematicidal activity

For nematicidal activity, a field-selected *M. javanic*a population from Barcelona, Spain was used. Nematodes were maintained on tomato plants (*S. lycopersicum* L. var. *Marmande*) cultivated in pot cultures within environmentally controlled growth chambers at 25 ± 1°C and >70% relative humidity. Egg masses of *M. javanica* were collected from infected tomato roots two months after seedling inoculation. Second-stage juveniles (J2) were obtained by incubating the egg masses in a water suspension at 25°C for 24 h.

#### 2.4.1 *In vitro* effects on juveniles J2

Bioassays were performed in 96-well plates (BD Falcon, San Jose, CA, USA), as previously described (Andres et al., 2017). Each well contained 100 J2 and 95 mL of water. Test solutions were prepared at 10 mg/mL in 0.2% Tween 20 in DMSO. The treatments and controls were added to each well, resulting in an initial concentration of 0.5 mg/mL. Nematicidal activity data are presented as the percentage of dead J2 (mortality was checked by transferring paralyzed juveniles to distilled water for further examination after 5 h–6 h), corrected according to Scheider–Orelli’s formula. Probit analysis was used to calculate the effective lethal doses (LC_50_ and LC_90_) by serial dilutions (five to eight concentrations), and each concentration was replicated four times. Thymol (Sigma-Aldrich) was used as a positive control, with effective doses (LD_50_ and LD_90_) of 0.143 (0.131–0.143) and 0.219 (0.209–0.230) mg/mL, respectively (Andres et al., 2012).

#### 2.4.2 *In vitro* effects on egg hatching

Three uniform-sized egg masses were washed with sterilized distilled water and placed in a 96-well plate containing treatments at minimum lethal concentrations (MLCs) and control samples, as described for the *in vitro* effects on juvenile J2. Each experiment was replicated four times. After the five-day incubation period, the hatched J2s were counted, and the test solutions were replaced with sterilized distilled water. Egg masses were monitored weekly and hatched J2s were counted for each immersion time (Andres et al., 2012). The data were transformed using Log10 (x + 1) and subjected to ANOVA analysis, with means separated by LSD at p <0.05. The relative suppression rate for each immersion time was calculated as follows: relative suppression rate (%) = (number of J2 in control − number of J2 in test solutions)/number of J2 in control × 100.

#### 2.4.3 *In vivo* effects on tomato plants

The EO treatments were tested at two concentrations: minimum lethal concentration (MLC) and an additional 1:2 dilution (0.03 mg/mL and 0.015 mg/mL) in 1% ethanol. The solutions were then mixed with sandy loam soil and transferred to plastic pots. Each pot was inoculated with *M. javanica* eggs and incubated for five days in a growth chamber under specific conditions. The control pots contained 1% ethanol. After the incubation period, one-month-old tomato seedlings were transplanted and maintained under the same conditions. Each experiment was repeated four times. At harvest, after 60 days, the roots were processed to determine nematode infectivity, infection frequency (IF), egg production, and multiplication rate (MR). The data were transformed using Log10 (x + 1) before performing an analysis of variance, with means separated by LSD at p <0.05. The relative reduction rate of nematode population reproductive traits (compared to the control) was calculated.

### 2.5 Phytotoxic activity

Phytotoxity experiments were conducted using ryegrass (*L. perenne*) and tomato (*S. lycopersicum*) seeds (40 seeds per test) in 12-well microplates (with three replicates) following the procedure described by Ruiz-Vasquez et al. (2022). Essential oil was tested at 0.4 mg/mL and 0.1 mg/mL concentrations in each well. Juglone (Sigma) served as a positive control at 0.1 mg/mL concentration, leading to 100% germination inhibition. The germination process was monitored for seven days, and at the end of the experiment, the leaf (for ryegrass) or root (for both species) lengths of 25 randomly selected plants were measured and digitalized. Non-parametric analysis of variance (ANOVA) was performed on the root/leaf length data.

### 2.6 Molecular docking

The membrane protein involved in coupled K^+^/Cl^-^ movement in the plasma membrane, the potassium chloride co-transporter KCC3 (PDB ID 7D90), was chosen as the target for molecular docking (Chen et al., 2019). It was downloaded from the PDB (Protein Data Bank) website and imported into Discovery Studio software. The heteroatoms and water molecules were removed from the structure. The binding sites were determined and visualized. Molecular docking was performed using PyRx 0.8 (Kitete et al., 2022; Ngbolua et al., 2022).

Ligand structures were downloaded in SD format and isomeric smiles of each compound were used for ADMET (pharmacokinetic properties) using pKCSM (Ngouana et al., 2011; Ferreira et al., 2015). Thiirane, 2-octyl-, 1-oxide, surfactant (Pubchem ID 85766188), approved by the U.S. Food and Drug Administration (FDA) commonly used against worms, was used as a positive control.

## 3 Results

### 3.1 Essential oil yield and chemical composition

The essential oil extracted from the stem bark of *D. gossweileri* was dark yellow, with an average oil yield of 0.55% (v/w), calculated on a dry weight basis. GC–MS analysis identified five constituents, representing 100% of the total. The main constituent is benzyl isothiocyanate (96.23%). **Table 1** lists the chemical constituents, retention time (min), molecular formula, and percentage of area.

**Table 1:** Chemical composition of *Drypetes gossweileri*.

Compound	Formula	% Area	Retention Time (min)
α-Pinene	C_10_H_16_	0.40	3.781
Benzyl chloride	C_7_H_7_Cl	0.74	4.955
Benzyl isocyanate	C_8_H_7_NO	1.25	6.948
Benzyl cyanide	C_8_H_7_N	1.38	7.297
Benzyl isothiocyanate	C_8_H_7_NS	96.23	12.250

### 3.2 Biocidal effects

The biocidal effects of EO against target species (insects and nematodes) are shown in **Table 2**. All the insect species tested were significantly affected by the oil according to their feeding adaptations (the polyphagous *S. littoralis* and *M. persicae* were less affected than the oliphagous *R. padi*) with efficient doses (EC_50_) of 29.4 μg/cm^2^, 14.744 μg/cm^2^, and 8.3 μg/cm^2^, respectively.The EO was very effective against the nematode *M. javanica*, with LC_50_ and LC_90_ values of 0.007 (0.006–0.0072) mg/mL and 0.0113 (0.0111–0.0116) mg/mL, respectively and more effective than the positive control thymol (LD_50_ and LD_90_ of 0.143 mg/mL and 0.219 mg/mL).

**Table 2:** Biocidal effects of *Drypetes gossweileri* essential oil against insect pests (antifeedant effects on *Spodoptera littoralis*, *Myzus persicae*, and *Rhoplalosiphim padi*) and the nematode *Meloidogyne javanica* (% mortality of juveniles J2).

Target	Dose	Activity (%)	EC_50_ ^e^/LC_50_ ^f^	LC_90_ ^f^
***S. littoralis***	100^a^	87.27 ± 11.32^c^	29.36^a^ (19.81–43.54)	
***M. persicae***	100^a^	ml100^c^	14.74^a^ (11.49–18.91)	
***R. padi***	100^a^	100^c^	8.31^a^ (6.10–11.32)	
***M. javanica***	1,000–15^b^	100^d^	7.0^b^ (6.0–7.2)	11.3^b^ (11.1–11.6)

a Dose in μg/cm^2^.

b Dose in μg/mL.

c Feeding/Settling Inhibition (%FI/%SI).

d Mortality of J2 (%).

e Efficient dose to give 50% inhibition (Feeding/Settling) and 95% confidence limits (CL).

f Lethal doses to give 50% or 90% mortality and 95% confidence limits (CL).

**Table 3** shows the egg hatching inhibitory activity *in vitro D. gossweileri* EO, tested at the minimum lethal concentration (MLC, 0.03 mg/mL) and lower (0.015 mg/mL). EO significantly reduced the number of J2s hatched at each incubation time (95%–100% and 89%–100% total suppression at 0.03 mg/mL and 0.015 mg/mL, respectively). After five days of incubation (day 0), the EO at 0.03 mg/mL gave a strongly inhibited egg hatching (94.7%), which increased with time, resulting in a total reduction (100%) at 14 days. Similarly, after five days of incubation (day 0), 0.015 mg/mL moderately suppressed egg hatching for 7 days (89.5%–86.7%) after 14 days to give a total inhibition (99%–100%). Therefore, the two tested concentrations strongly inhibited the hatching of J2 *M. javanica* from eggs with a final rate of suppression >96%.

**Table 3:** Effects of *Drypetes gossweileri* essential oil against *Meloidogyne javanica* egg masses with time. The oil was tested at the minimal lethal concentration on J2 (MLC mg/mL) and a 1:2 dilution.

EO [mg/mL]	Hatched juveniles and relative hatch suppression rate (%) with time^1^										
	0		7		14		21		28		Total SR
**Control**	229.70 ± 16.72^,a^		674.5 ± 62.1^a^		1626.0 ± 122.7^a^		304.0 ± 68.9^a^		57.25 ± 11.60^a^		
**[0.03]**	12.1 ± 3.4^b^	94.7^3^	30.7 ± 4.9^b^	95.4	2.7 ± 0.8^b^	99.8	0^b^	100	0^b^	100	99
**[0.015]**	24.0 ± 3.1^c^	89.5	86.7 ± 9.4^c^	89.8	21.5 ± 4.5^c^	98.8	3.25 ± 0.6^c^	99	0^b^	100	96

^1^Time 0: Five days of incubation. Subsequent times: number of days of immersion in water after time 0.

^2^Values are mean ± standard error of hatched juveniles from three egg masses/four replicates. Values within the same column followed by different letters are significantly different, Least Significant Difference (LSD) test (p <0.05).

^3^Each value represents the percent relative suppression rate (SR: number of J2 in control − number of J2 in treatment)/number of J2 in control × 100).

**Table 4** shows the effects of the essential oils on the reproduction of *M. javanica*. EO tested at the MLC (0.03 mg/mL) and lower (0.015 mg/mL), significantly reduced the reproductive traits of the population of *M. javanica* (egg mass/plant, eggs/plant) and the infection frequency (IF) and multiplication rate (MR) indexes compared to untreated control of tomato plants in pot experiments. EO at 0.03 mg/mL (MLC) resulted in a total reduction (100%) in the number of egg masses of nematodes, IF, and MR. EO at 0.015 mg/mL caused a high suppression rate (≥98%) of the number of eggs per plant. The growth of the tomato plants was not influenced by these treatments (data not shown). These *in vivo* results confirmed the strong *in vivo* effects of this essential oil on J2 mortality and hatching in tomato plants.

**Table 4:** *In vivo* effects *Drypetes gossweileri* essential oil on reproductive traits of *Meloidogyne javanica* in tomato plants, 60 days post-inoculation, with 2,000 eggs per plant, maintained in a growth chamber.

EO [mg/mL]	Egg masses/plant^1^	RS^2%^	Eggs/plant x100	RS^3%^	IF^4^	MR^5^
**Control**	462.75 ± 43.48 ^a^		2,990 ± 229 ^a^		0.2361	155.25
**[0.03]**	0^c^	100	0^c^	100	0	0
**[0.015]**	10.40 ± 2.08 ^d^	98	38 ± 10 ^d^	99	0.0052	1.91

^1^Values are mean ± standard error of 10 plants. Values within the same column followed by different letters are significantly different, Least Significant Difference (LSD) test (p <0.05). ^2^Relative suppression on eggs masses. ^3^Relative suppression on number of eggs per plant. ^4^Infection Frequency: egg masses per plant/egg inoculum. ^5^Multiplication Rate: eggs per plant/egg inoculum.

**Figure 1:**
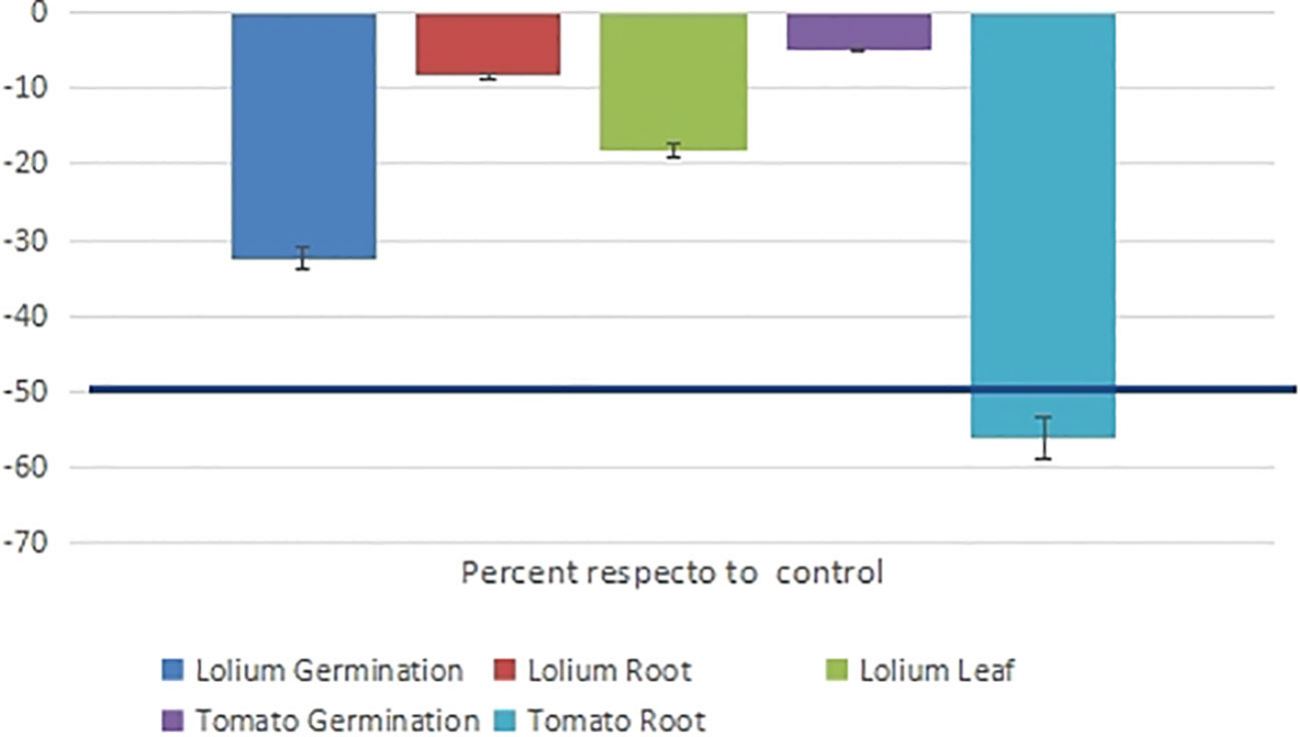
The phytotoxic effects (germination and growth inhibition at 7 days) of *Drypetes gossweileri* essential oil against the plant species *Lolium perenne* and *Solanum lycopersicum* were expressed as a percentage of the control.

The phytotoxic effects of *D. gossweileri* essential oil against ryegrass (*L. perenne*) and tomato (*S. lycopersicum)* seeds (**Figure 1**). The EO at 0.4 mg/mL, inhibited ryegrass germination (for up to 7 days). At 0.1 mg/mL, germination and root and leaf growth were moderately inhibited (32.4%, 8.4%, and 18.3% inhibition, respectively). At a dose of 0.1 mg/mL, which is 10 times higher than the LC_50_ calculated for the mortality rate of *M. javanica* J2, the germination of tomato was not affected (5% inhibition), but the root growth rate decreased by 56%.

### 3.3 Molecular docking

Molecular docking was performed for nematicidal effects, as this was the most important biocidal effect of the essential oil. PyRx 0.8, and ligands and macromolecules were visualized on Discovery Studio.

The binding affinities and H-bonds of *D. gossweileri* EO compounds with KCC3 are presented in **Table 5**. It can be noticed that Thiirane, 2-octyl-, 1-oxide, surfactant (PubChem CID 85766188) used as the positive control, has a binding affinity of −4.90 ± 0.23 kcal/mol, while Benzyl cyanide has −5.10 ± 0.28 kcal/mol followed by benzyl isocyanate −4.70 ± 0.14 kcal/mol.

**Table 5:** Binding affinity and H-bonds.

Compounds	Binding affinity (kcal/mol)	Hydrogen bonds			
		Count	Substrate	Distance (A°)	Sum of Vander Waals radii (A°)
Thiirane (Control)	−4.90 ± 0.23	1	GLN C:350	2.27	2.75
Benzyl isocyanate	−4.70 ± 0.14	1	GLU C:66	2.33	2.75
Benzyl cyanide	−5.10 ± 0.28	1	GLN C:350	2.20	2.75
Benzyl isothiocyanate	−4.60 ± 0.16	2	ARG B:294	2.78	2.75
			GLN C:97	2.90	2.75

**Table 6** shows the predictive results of the ADMET analysis obtained at http://biosig.unimelb.edu.au/pkcsm/prediction after uploading the compound smiles (Daina et al., 2017). Benzyl cyanide showed the best binding affinity (−5.10 ± 0.28 kcal/mol) (**Table 5**) and also has an interesting predictive result on ADME-T (**Table 6**).

**Table 6:** ADMET profile.

Parameters		Benzyl isothiocyanate	Benzyl isocyanate	Benzyl cyanide	Thiirane, 2-octyl-, 1-oxide, surfactant
Absorption and Distribution	BBB	0.45	0.078	0.17	0.732
	HIA	94.774	96.135	95.113	95.193
Metabolism	CYP2D6 substrate	No	No	No	No
	CYP3A4 substrate	No	No	No	No
Excretion	Total Clearance	0.305	0.831	0.319	1.507
	Renal OCT2 substrate	No	No	No	No
Toxicity	Oral Rat Acute Toxicity (LD_50_)	2.254	1.98	1.935	1.804
	Hepatotoxicity	No	No	No	No

**Figure 2:**
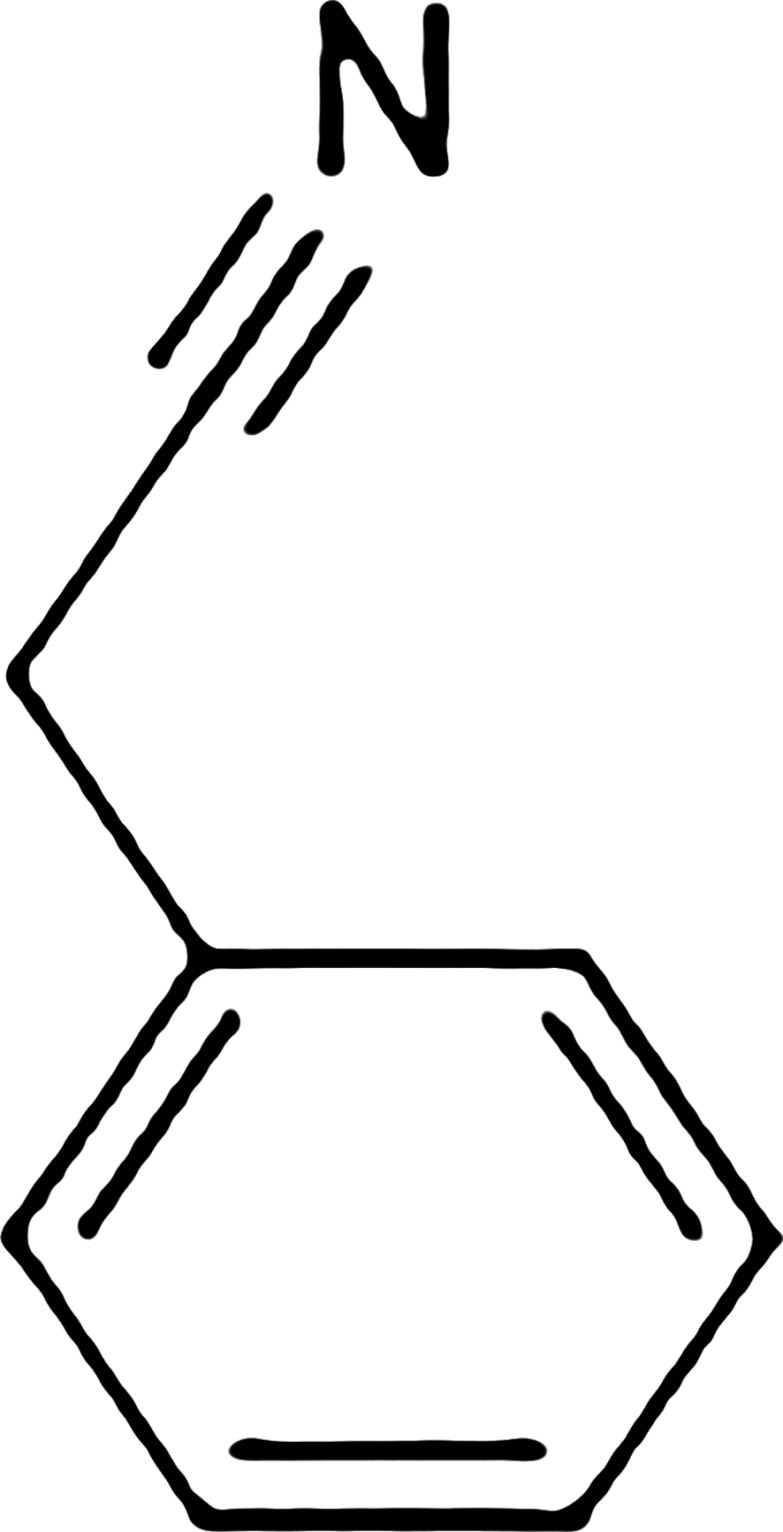
Structure of benzyl cyanide.

**Figure 3:**
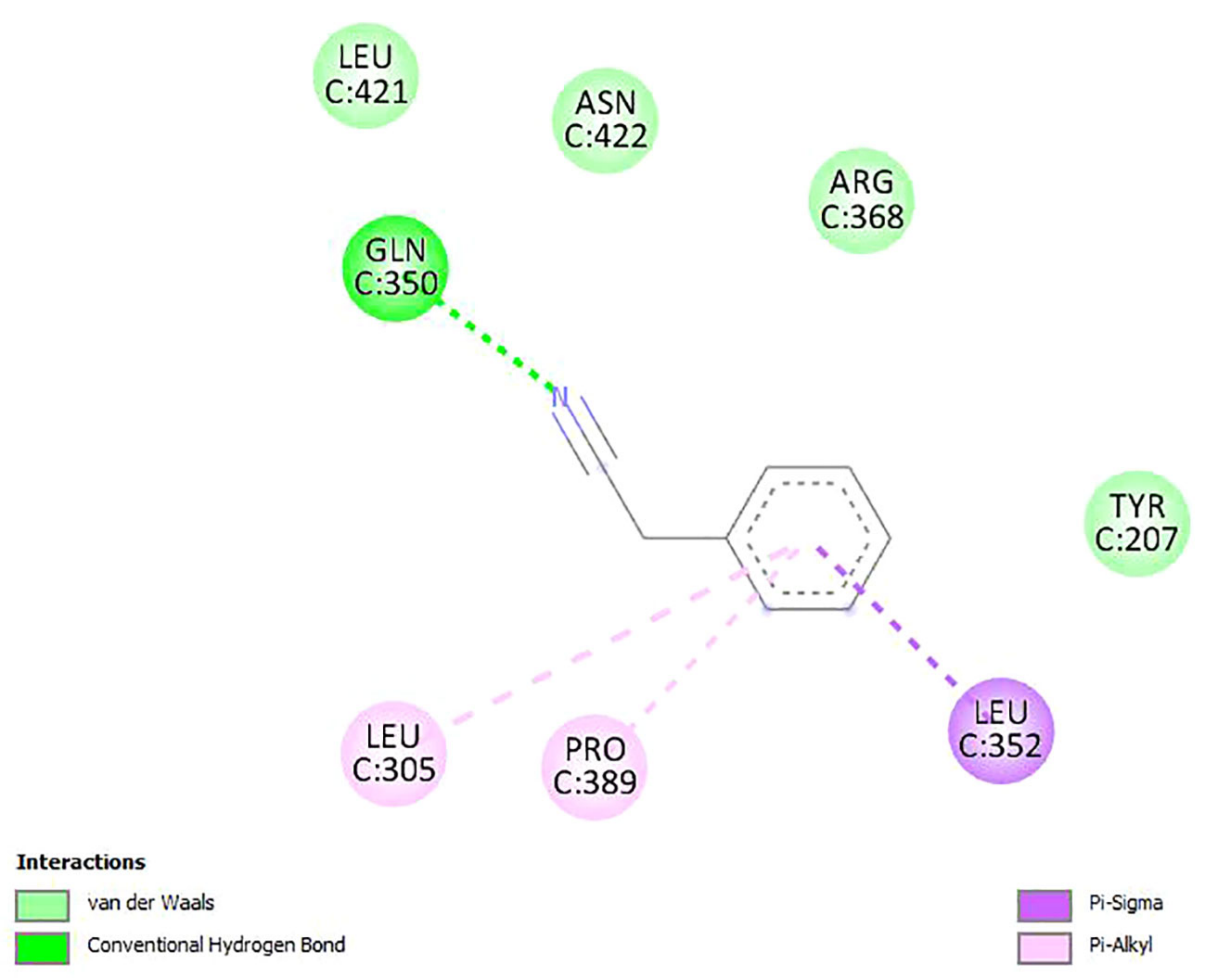
Molecular docking between benzyl cyanide and potassium chloride cotransporter KCC3.

**Figure 4:**
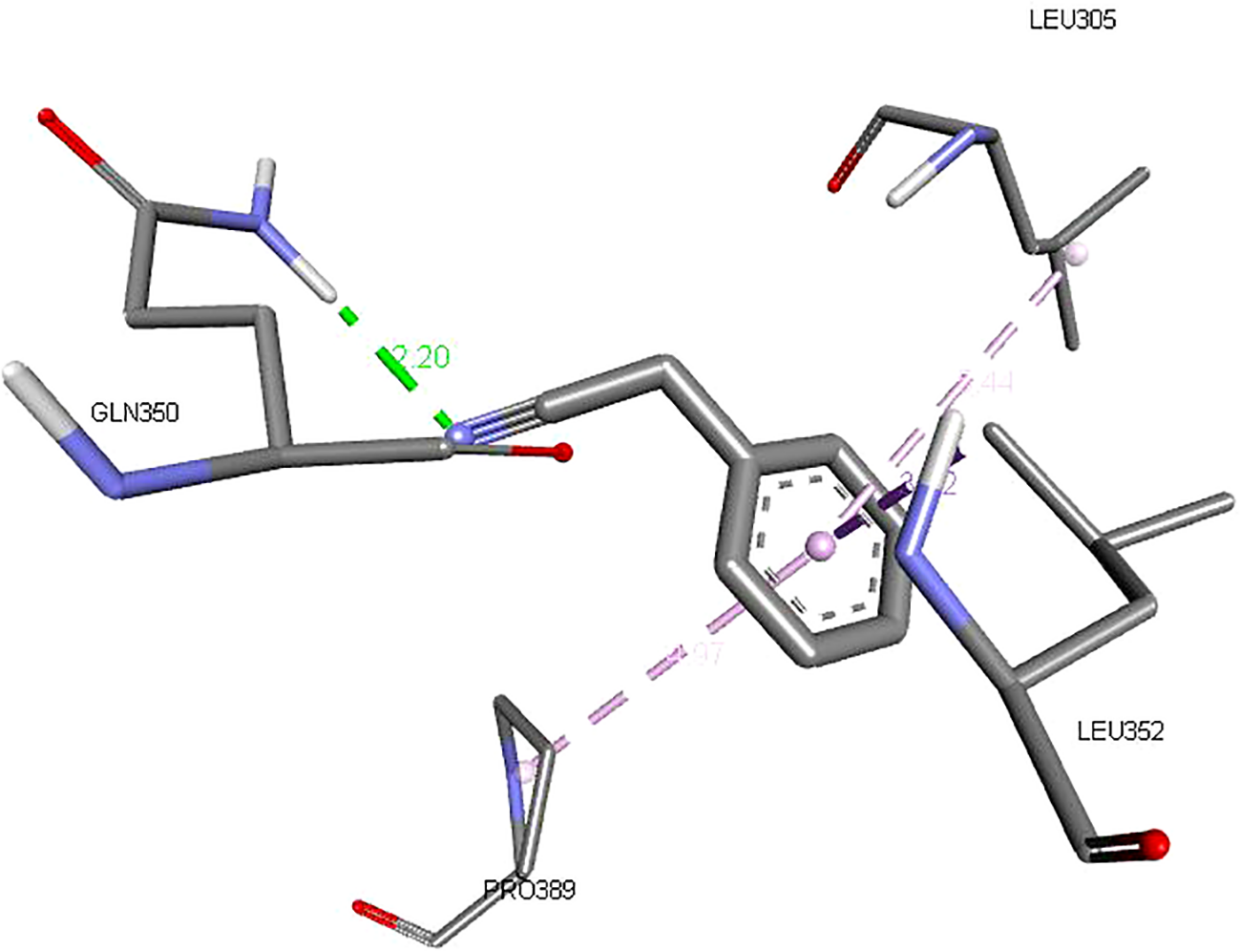
The ligand interaction distance.

**Figures 2**–**4** show the structure of benzyl cyanide, molecular docking, and interaction distance. The identification of the H-bond type (green) and ligand interaction distance are presented in **Figure 4**. It can be observed that the conventional H-bond is established on GLN C:350 as the substrate (amino acid).

## 4 Discussion

The essential oil yield from *D. gossweileri* leaves was 0.55%. Yields of 0.2% and 0.29% have been previously reported for bark essential oils (Eyele Mvé-Mba et al., 1997). The composition of the essential oil of *D. gossweileri* leaves from Kisangani was largely dominated by benzyl isothiocyanate followed by benzyl cyanide. Similar results have been reported for the composition of *D. gossweileri* stem bark EO from Cameroon (Ndoye Foe et al., 2016), with benzyl isothiocyanate as the main compound, followed by benzyl cyanide. However, benzyl cyanide (73.7%) was the main compound in the *D. gossweileri* bark EO from Gabon (Agnaniet et al., 2003). Benzyl isothiocyanate has been reported in bark essential oil (Eyele Mvé-Mba et al., 1997; Voundi et al., 2015). These variations may be related to plant origin, plant parts used, or the distillation process that causes thermal degradation of glucosinolates (GL). Previous studies showed the presence of 4-hydroxybenzyl (12.5%) and benzyl GL (87.5%) in the bark and 4-hydroxybenzyl GL in the seeds of *D. gossweileri* (Montaut et al., 2017).

In this study, *D. gossweileri* leaf essential oil, rich in benzyl ITC, was used as an insect antifeedant with effective doses that varied with the insect species and feeding adaptation (lower effect against the polyphagous *S. littoralis*: 29 μg/cm^2^, and *M. persicae*: 15 μg/cm^2^, and stronger against the oliphagous *R. padi*: 8 μg/cm^2^). Similarly, previous studies have shown that two generalist (*S. frugiperda* and *Trichoplusia ni*) lepidopterans were adapted to feed on allyl and benzyl isothiocyanate-containing crucifers because these compounds are metabolized by glutathione transferase present in the generalist and absent in the specialist insect species (*Anticarsia gemmatalis*) (Wadleigh and Yu, 1988). Isothiocyanates (ITCs), released from Brassicaceae plants after hydrolysis of glucosinolates, are known for their negative effects on insect herbivores (Hopkins et al., 2009) and have been proposed for isothiocyanate-based biofumigation, being aliphatic isothiocyanates stronger fumigants than the aromatic ones because of their lower volatility (Matthiessen and Shackleton, 2005). Therefore, the lower volatility of benzyl isothiocyanate could be advantageous for formulation purposes.

The essential oil of *D. gossweileri*, rich in benzyl isothiocyanate, was lethal to *M. javanica* J2. At MLC concentrations and below (0.03 mg/mL–0.015 mg/mL) this oil strongly inhibited egg hatching *in vitro*, and in soil treatment (*in vivo*) caused strong suppression (100%–98%) of nematode egg masses, egg production, infection frequency and multiplication rate in tomato plants. Glucosinolates and isothiocyanates (such as benzyl isothiocyanate) have been reported in the literature as having nematicidal properties (Wu et al., 2011). Soil biofumigation with ITC-producing *Brassiccaceae* has been proposed for the control of *Meloidogyne* species in the context of Integrated Pest Control Management strategies (Ntalli and Caboni, 2017). Essential oil from the seeds of *Carica papaya* showed *in vitro* nematicidal activity against *Caenorhabditis elegans* and *M. incognita*, with benzyl isothiocyanate being responsible for the activity (Nagesh et al., 2002). Benzyl isothiocyanate showed strong *in vitro* nematicidal activity against *M. incognita* J2 (LD_50 =_ 15.21 μM) (Lazzeri et al., 2004). The infectivity of treated (0.01 mM) *M. incognita* juveniles on soybean (*Glycine max*) was significantly reduced, and egg production was almost eliminated by 0.03 mM benzyl isothiocyanate (Zasada et al., 2009). Reproduction and infectivity of *M. incognita* were suppressed when juveniles (J2) were exposed to 0.03 mM benzyl isothiocyanate for 2 h prior to inoculation with either *C. annuum* or *G. max* (Masler et al., 2010). However, the mode of nematicidal action of these isothiocyanates remains unknown.

It has been suggested that isothiocyanate interacts with cellular proteins and amino acids through oxidation reactions. Potassium ion homeostasis is reported in the literature to be a key factor in the maintenance of many vital functions in living beings. Indeed, the transport of this ion across the biological membrane is coupled to that of the chloride ion and ensured by cation-chloride protein co-transporters. It is involved in the change of the cellular response to the neuromodulator gamma amino butyric acid (GABA), which is the basis for the transition from a depolarizing effect to a hyperpolarizing effect, with the consequent inhibition of neurotransmission (Lesage et al., 2000). This bioelectric phenomenon, necessary for neuron maturation, was exploited in this study as a pharmacological target of the compounds contained in the essential oil of *D. gossweileri*. Thus, in the present study, we demonstrated through molecular modeling that the bioactive compounds of this essential oil form a thermodynamically stable complex with the protein receptor KCC3 (ΔG <0) in the same order of importance as that of the positive control (thiirane). In addition, all these compounds (as well as the positive control) formed a hydrogen bond with the receptor at the protein C subunit, demonstrating the synergistic effect of the bioactive compounds of this essential oil. Thus, the interaction of these compounds with the KCC 3 receptor would result in the inhibition of neuronal maturation (synaptic inhibition) via modification of the electrochemical properties of the biological membrane and thus the membrane transport of chloride ions, hence the increase in neuronal excitability, which could justify the (neuro) toxic effects observed in nematodes under our experimental conditions. The mechanism proposed in this study is derived from the principles of previous experiments with *C. elegans*, a nematode used as an experimental model/system in molecular biology to understand cell differentiation phenomena (Leung et al., 2008). Thus, neuronal maturation in *C. elegans* under the physiological action of GABA via the co-transporter KCC is a fundamental property common to all nematodes (including *M. javanica*). This biological property, being well preserved in this taxonomic group during ontological evolution, allows us to extrapolate the knowledge on GABA receptors to root-knot nematode species (*M. javanica)* as a pharmacological target of interest, under the postulate of the uniformity of biological processes that are the basis of cell function.

Molecular docking-based understanding of the bioactivity of EOs is a novel tool for the selection of natural products and discovery of biopesticidal leads. For example, *in silico* analysis suggested a higher binding capacity of geraniol, β-terpineol, citronellal *l*-limonene, γ-terpinene to ODR1 (odorant response gene 1) complex due to extensive H-bonding, hydrophobic, and p-alkyl interactions among a selection of target proteins (cytochrome c oxidase subunit 1, AChE, Hsp90, ODR1, ODR3, neuropeptide GPCR, and CLAVATA3/ESR (CLE)-related) of the nematode *M. incognita*, which was confirmed *in vitro* (Kundu et al., 2021). Therefore, *in vitro/in vivo* effects and computational bioefficacy analyses of EO chemical profiles can provide useful insights into the potential of EOs as bionematicides.

In light of our results, mustard EO from the leaves of the medicinal plant *D. gossweileri*, characterized by benzyl isothiocyanate (BITC), could be used as a safe alternative to synthetic chemical products. Therefore, ITCs are potentially toxic to humans. Specifically, BITC has been shown to cause genotoxicity, chromosomal aberrations, and DNA damage (Masutomi et al., 2001). BITC was orally administered (200 mg/kg daily for 4 weeks) to rats, with alterations in hematological parameters. However, BITC has been proposed as an anticancer agent and is considered safe for consumption (Dinh et al., 2021). Therefore, essential (mustard) oils from tropical forests are credible alternatives to synthetic chemical products. The Democratic Republic of Congo, a sanctuary of medicinal plants, is therefore a source of biologically active chemical compounds that can be exploited as biopesticides. To prevent deforestation and reduce human pressure on natural forests, large-scale cultivation (domestication) of this wild plant should be considered in a project to create a productive ecosystem for a sustainable supply of raw materials. Indeed, in the context of biodiversity loss linked to human activities and environmental factors (climate change), safeguarding natural resources must involve the implementation of efficient management and conservation strategies. A processing unit for this medicinal and aromatic plant can also facilitate socioeconomic development through an industrial agronomic initiative. The domestication of wild plants helps fight deforestation and climate change by creating a carbon sink to reduce greenhouse gases, as recommended by the Kyoto Protocol (Atangana et al., 2014).

## 5 Conclusion

The aim of the present study was to determine the chemical composition of the essential oil of a tropical plant, *D. gossweileri* S. Moore, and to evaluate its insecticidal and nematicidal effects against insect pests (*S. littoralis*, *M. persicae*, and *R.padi*), and the root-knot nematode *M. javanica*. GC/MS analysis of the essential oil of *D. gossweileri* indicated the presence of benzyl isothiocyanate as the major component followed by small amounts of benzyl cyanide, benzyl isocyanate, benzyl chloride, and α-pinene. ITC found in the EO is derived from the hydrolysis of the parent glucosinolate present in the leaf tissue. This mustard essential oil showed significant species-dependent insect antifeedant effects, strong *in vitro* and *in vivo* nematicidal activity, and low phytotoxicity against tomato plants. The major EO components (benzyl isothiocyanate, benzyl cyanide, and benzyl isocyanate) are believed to act by inhibiting the KCl cotransporter.

Mustard EO from the leaves of the medicinal plant *D. gossweileri* could be used as a safe alternative to synthetic chemical pesticides (insecticides and nematicides applied to the soil). Further research is needed to develop productive agroforestry systems for the sustainable production of *D. gossweileri* oil-based medicinal and biopesticidal ingredients along with optimized formulations.

## Data availability statement

The datasets presented in this study can be found in online repositories. The names of the repository/repositories and accession number(s) can be found in the article/supplementary material.

## Author contributions

JM: Investigation, Formal Analysis, Writing – original draft. MA: Formal Analysis, Investigation, Supervision, Writing – review & editing. EK: Investigation, Writing – original draft, Methodology. NK: Investigation, Methodology, Writing – original draft. DDT: Investigation, Writing – original draft, Formal Analysis. KN: Investigation, Methodology, Writing – original draft. DTT: Investigation, Writing – original draft, Methodology. OO: Investigation, Methodology, Writing – original draft. AG-C: Investigation, Writing – original draft, Funding acquisition, Resources, Supervision, Writing – review & editing. PM: Conceptualization, Investigation, Methodology, Resources, Supervision, Validation, Writing – original draft, Writing – review & editing.
